# Repair of a Bronchoesophageal Fistula Using a Pericardial U-Flap

**DOI:** 10.7759/cureus.85937

**Published:** 2025-06-13

**Authors:** Sammy Shihadeh, Christoph A Stephenson-Moe, Paul Vesco, M. Blair Marshall

**Affiliations:** 1 Clinical Sciences, Florida State University College of Medicine, Tallahassee, USA; 2 Department of Cardiothoracic Surgery, Sarasota Memorial Health Care System, Sarasota, USA

**Keywords:** bronchial fistula, bronchial repair, bronchoscopy, esophageal diseases, esophageal fistula, esophageal repair, esophagectomy, respiratory tract fistula

## Abstract

Bronchoesophageal fistula (BEF) is a severe complication of esophagectomy and is burdened by high mortality rates, which has scarce reporting in the literature. These fistulas are usually the result of a prior leak from esophagogastric anastomosis. The etiology of a BEF after esophagectomy can be multifactorial. BEF occurrence can be further complicated by a history of esophageal malignancy, predisposing the patient to fistula formation.

We present a 71-year-old male with a history of esophageal cancer, treated initially with neoadjuvant chemoradiation and an Ivor-Lewis esophagectomy five months later, discharged on post-operative day six, who had subsequent clinical symptoms, primarily respiratory in nature, two weeks later. Imaging and workup revealed a BEF. After the patient was admitted, he was taken to the operating room (OR) for initial lysis of adhesions and clearance of necrotic tissue and aspiration of secretions. For approximately the next month, every four to five days, he was taken back to the OR for endoluminal sponge vacuum-assisted closure (VAC) placement and replacement as well as additional therapeutic aspiration of secretions, which were often purulent. As the patient was critically ill, this was determined to be the best course of action in the initial stabilization of the BEF as a bridging measure until definitive surgical management could be intervened. This was done in order to promote initial healing of the fistula to optimize tissue for surgical treatment (i.e., supported by presence of granulation tissue). The patient recuperated between procedures in the intensive care unit (ICU). Ultimately, the patient underwent surgical repair and esophageal exclusion.

The patient was discharged on post-operative day 60 after recovery and continues seeing his primary care physician and the surgical groups who managed his care to assess for changes in symptoms and follow-up imaging. This case conveys the urgency of diagnosing and treating a BEF, demonstrating improved outcomes when surgically managed in a timely manner.

## Introduction

Bronchoesophageal fistula (BEF) is a rare clinical presentation that may originate from a variety of etiologies both benign and malignant, with esophageal cancer being the most common malignant cause. Five to 10% of patients with esophageal cancer may develop a BEF [1]. BEF may present with respiratory infections, dysphagia, hemoptysis, and is associated with a high mortality. The most common iatrogenic causes include prolonged endotracheal intubation, endoscopic procedures, and complications from esophagectomy, including anastomotic leak, as in our patient. Some mechanisms of fistula formation may involve insidious erosion of the walls between the esophagus and bronchus, such as in cancer-related causes, or iatrogenic/traumatic injury resulting in a direct perforation between the two. Incidence of BEF after esophagectomy is reported to be between 1.1 and 3% [2,3]. Surgical intervention is associated with fistula closure in 90-95% of cases, regardless of etiology [4]. If untreated, a BEF can result in recurrent pneumonia from aspirated gastric contents, can eventually produce pulmonary fibrosis, bronchiolitis, and pneumonitis if chronic. In severe situations, sepsis and death may occur in the acute setting [5,6].

## Case presentation

A 71-year-old male with a past medical history of esophageal cancer (staged T3N2MX) was treated with neoadjuvant chemoradiation and subsequent esophagectomy by the surgical oncology service five months later. The patient presented two weeks following discharge with persistent cough and dyspnea. For the few days prior to admission, the patient experienced worsening shortness of breath with minimal exertion, thin phlegm production, congestion, and intermittent wheezing. He denied chest pain, abdominal pain, and hemoptysis. Since his esophagectomy, he had been on a liquid diet and denied dysphagia. He experienced occasional nausea, early satiety, and post-tussive emesis. The patient's past medical history includes hypertension, gastroesophageal reflux disease (GERD), asthma, and paroxysmal supraventricular tachycardia (SVT) treated with cardiac ablation four years earlier. Pertinent medications that the patient is currently taking include metoprolol 25 mg twice daily, odansetron 4 mg for nausea as needed, and pantoprazole 40 mg once daily. He consumes two alcoholic drinks per day and has a 10 pack-year history of smoking, but quit smoking about 40 years ago.

**Figure 1:**
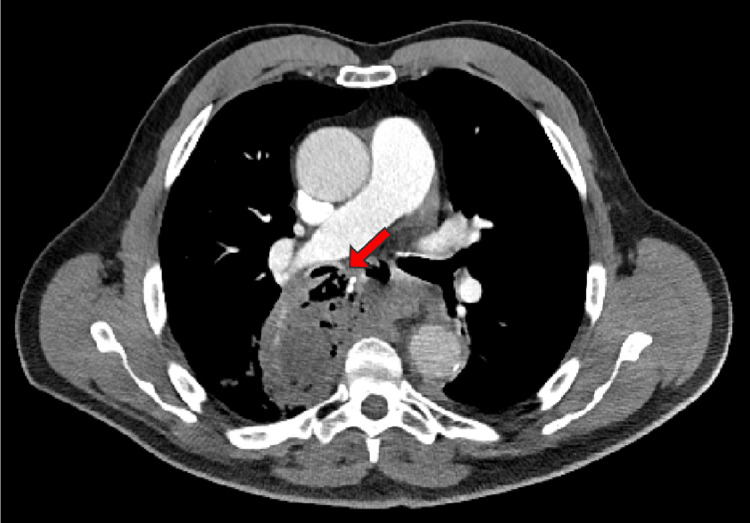
Axial view computed tomography (CT) of the thorax demonstrating the bronchoesophageal fistula (BEF) (indicated by the red arrow)

**Figure 2:**
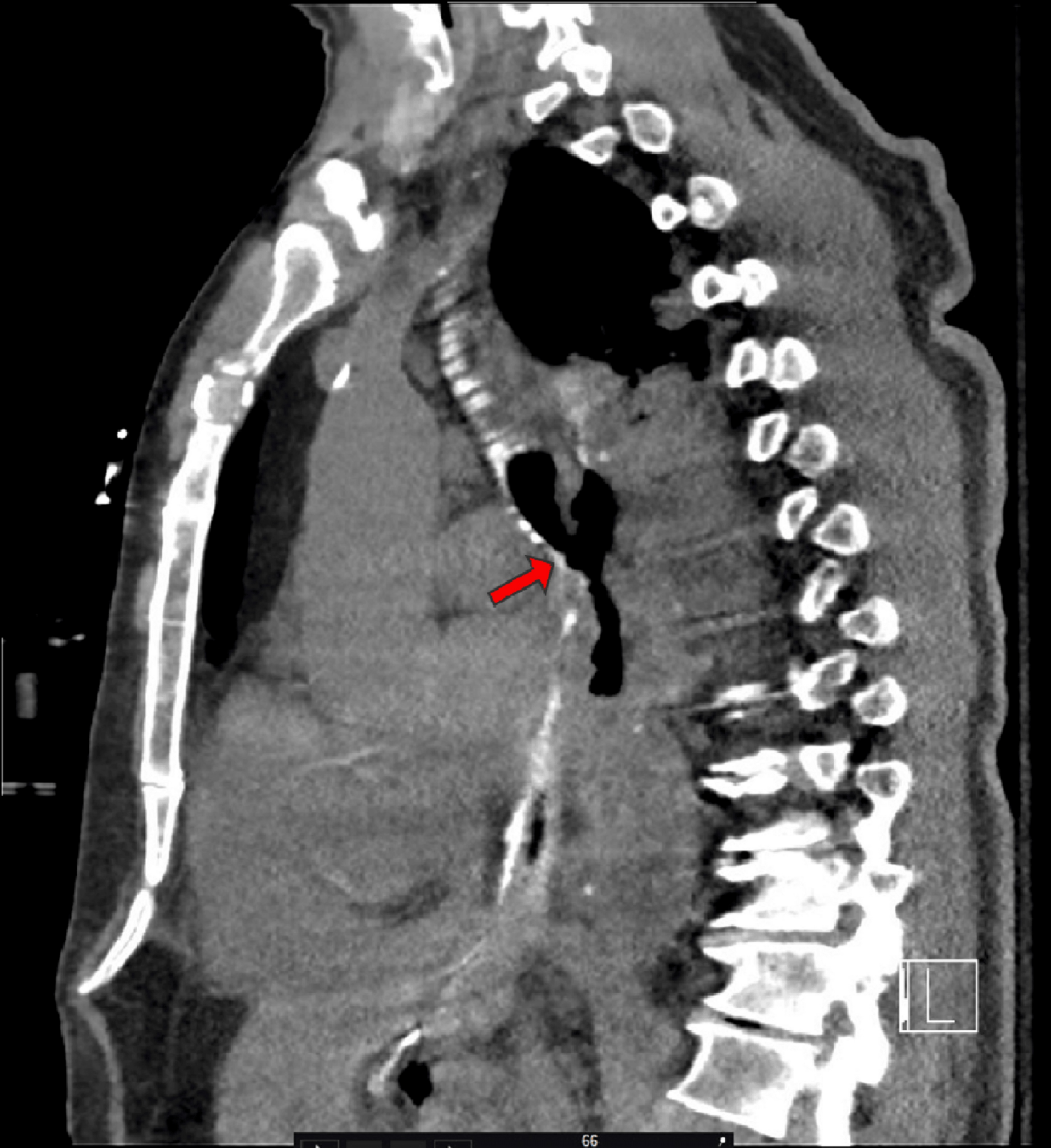
Sagittal view computed tomography (CT) of the thorax demonstrating the bronchoesophageal fistula (BEF) (indicated by the red arrow)

**Figure 3:**
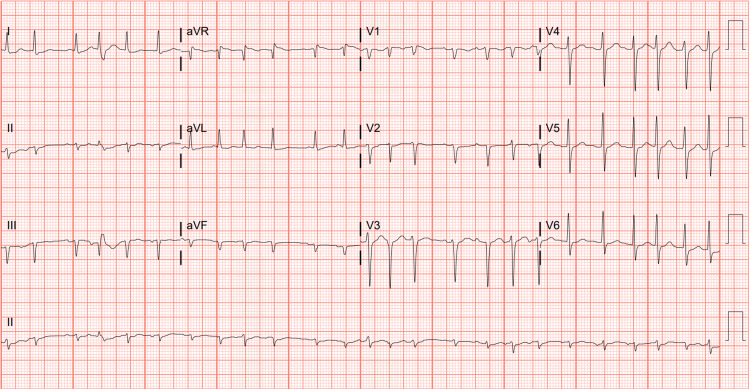
Electrocardiogram (ECG) demonstrating supraventricular tachycardia (SVT) with ventricular premature complexes now present; an old inferior infarct is evident on the ECG.

Differential diagnoses included pneumonia (i.e., community-acquired or aspiration), recurrence of esophageal cancer, asthma exacerbation, and myocardial infarction (MI). MI, being the most important diagnosis, was ruled out with a normal electrocardiogram (ECG) and troponin of 13 ng/L. A troponin level of 13 ng/L is generally considered within the normal range, but it's important to consider other factors. High-sensitivity troponin tests, like the one measuring 13 ng/L, are more sensitive than older troponin tests, allowing for earlier detection of heart damage. While a level of 13 ng/L is not typically considered a cause for major alarm, it's crucial to interpret the results in conjunction with the individual's symptoms, medical history, and other diagnostic findings. A computed tomography (CT) scan demonstrated a right-sided BEF (Figures 1, 2), which was ultimately surgically repaired once the patient was stabilized. Aspiration pneumonia may have also complicated the patient's presentation due to the aberrent communication between the esophagus and bronchus. The patient initially presented critically ill requiring 100% FiO_2_. Vitals during admission included a temperature of 100.7 °F, heart rate of 126 beats per minute, respiratory rate of 20 breaths per minute, and blood pressure of 132/81. An ECG taken during admission also demonstrated an episode of SVT (Figure 3). He was managed with an endoluminal vacuum-assisted closure (VAC) sponge and airway stent. During this management, he was found to have persistent cancer in the remaining portion of his esophagus. Once stable, he underwent a completion esophagectomy, pericardial flap repair of the bronchial fistula, omental flap, and cervical esophagostomy for feeding to allow the surgical portion of the esophagus to heal. Final margins were negative for malignancy.

Surgical techniques and outcomes

A right-sided posterolateral thoracotomy incision was made, and the fifth intercostal space was entered. The pleural space was found to be completely fused. The extrapleural plane was accessed and taken down in order to facilitate the intrapleural plane. The parietal pleura was fused to the visceral pleura as well, and so these dense pleural adhesions to the lung parenchyma were mobilized. It was dissected posteriorly and then both superiorly and inferiorly to identify the fistula. The posterior aspect of the esophagus and gastrointestinal conduit were dissected out to isolate the bronchial fistula. The gastric conduit was dissected distally as far as possible, then divided with a linear stapler. The defect measured approximately 2.2 cm x 1 cm. A U-shaped flap of pericardium, approximately 4 cm x 3 cm, was created and then doubled on itself to provide mobile robust tissue for the reconstruction; it was created along the left atrium and parachuted down over the remaining bronchial defect. Once secured, reconstruction was assessed for any air leaks. A portion of the omentum from the remaining gastric conduit was mobilized and covered over the defect. The chest was closed and a cervical esophagostomy was performed.

During the operation, lung isolation was challenging because of hypoxia and hypercarbia. In order to ventilate the right lung but reconstruct the bronchus intermedius, manipulating the stent with a retrograde small Foley balloon and then later during the procedure through a Fogarty catheter (sized 4 French) from above was conducted. The patient recovered without acute complications post-operatively and was discharged for future care in rehabilitation. The patient received physical rehabilitation and sessions with speech-language pathology for cognition, swallowing, and communication. He currently attends follow-up visits with his primary care physician and cardiothoracic surgeon.

## Discussion

Acquired aero-digestive fistulae almost always occur in adulthood, resulting from malignancy approximately half of the time. Although associated with significant morbidity, 10% of patients may be asymptomatic [7]. A BEF frequently occurs on the right thoracic side with the distal esophagus due to the right bronchus’ vertical orientation and close proximity to the esophagus [8]. Esophagram is the most sensitive testing to determine fistulae with barium being the contrast of choice as gastrografin may cause pulmonary edema and respiratory failure if it enters the lungs via the bronchial fistula [9,10].

Seventy-seven percent of BEFs caused by malignancy are secondary to esophageal cancer and 16% are secondary to advanced-stage lung cancer [11]. Esophagectomy was described as being one of the most common iatrogenic causes of BEF as well. It may be difficult to ascertain the primary etiology if both a history of esophageal cancer and surgical procedures have been performed. Each risk factor may insidiously, gradually contribute to the BEF formation, as demonstrated by our patient who had simultaneously presented with an additional esophageal malignancy and recent esophagectomy.

Though the incidence is as low as 0.28% to 3%, BEF can be a fatal complication, and gastric conduit necrosis and anastomotic leakage are the most common reasons for it; anastomotic leak(s) or ischemic necrosis of the gastrointestinal conduit created during the initial esophagectomy is the prevailing iatrogenic etiology in the formation of a BEF, which occurred in our patient, since refluxed gastric acid and infection can erode through the esophagus and into the bronchus [12,13]. The use of a pericardial flap was in part due to its convenient location near the fistula, further reinforced by a segment of omentum from the gastrointestinal conduit to ensure a complete seal after a pulmonary leak, as observed intraoperatively. Pericardial fat pads can also be manipulated in this practice, but the pericardium itself has been shown to be superior in surgical outcomes [14].

Esophageal stenting is the most common non-surgical intervention used to treat BEF. However, surgical repair provides better outcomes than stenting for patients desiring definitive treatment. Stents placed as a temporizing measure for patients only desiring palliative measures have shown that stenting alone is sufficient, in particular double-stenting [15,16]. Stents can be placed via flexible endoscopy or rigid bronchoscopy depending on the location and complexity of the fistula [17]. Maintaining patency of the trachea can be facilitated with a silicone T-tube, which has been shown to improve surgical outcomes [18]. Although open thoracotomy with primary repair is the most common means of intervention, video-assisted thoracoscopy has also been used in the treatment of BEF, including lobectomy or pneumonectomy if indicated intraoperatively [19].

Long-term outcomes of surgical repair of BEF are controversial. A study demonstrated 100% survival in patients at five years and an average survival time of 124 months [20]. Other studies include a retrospective study pertaining to a small sample size of 14 patients demonstrated a 14% mortality rate and another study demonstrated a 60% mortality rate from a sample size of 11 due to factors such as sepsis and organ failure [15,21]. A literature review demonstrated that the most common post-operative complications include anastomotic leak/stricture, necessity of trans-anastomotic feeding tube, dysphagia, gastroesophageal reflux (GER), and metaplasia [22]. Stricture formation may occur due to ischemia caused by disruption of the esophageal blood supply when manipulated during surgery [23]. A meta-analysis demonstrated no clinically significant difference in outcomes based on surgical technique, such as the open thoracotomy or thoracoscopic approach [24]. However, data is limited and equivocal as to only a few case reports/series that have been performed on the topic of surgically managing BEF after esophagectomy. 

Additionally, in a case series of four patients similarly presenting with bronchoesophageal fistula, surgical intervention in the form of thoracotomy was performed in three patients and cervicotomy in one patient [25]. Cervicotomy is a surgical incision made in the neck, specifically in the area between the collarbone and the chin. It is commonly used for accessing the thyroid gland, parathyroid glands, or other structures in the neck, such as for thyroidectomy or parathyroidectomy. The incision is typically a collar-transverse incision, which allows for a direct view of the desired surgical site. The approach discussed involved excision of the fistula with primary closure of the defects in the esophagus and bronchus, and placement of a pleural flap. Additional surgery was necessary in three of the patients, with a left pneumonectomy in another patient. Compared to our case, the initial approach was equivalent, but without placement of a pleural flap. 

However, our operation was complicated due to the varied pathological anatomy due to complications of esophageal malignancy, radiated tissues, and prior esophagectomy. This demonstrates that the etiology heavily influences initial management, both temporizing and definitive, in the patient presenting with BEF. These cases and their results help to support the efficacy of surgical intervention in the treatment of BEF.

Surgical treatment appears to be the most effective in the acute setting to stabilize the patient, as these patients often present septic and require emergent intervention. Endoluminal vacuum-assisted closure (E-Vac) therapy is a safe and effective treatment for upper gastrointestinal leaks and should be considered alongside more established therapies. Further research is now needed to understand the mechanism of action and to improve the ease with which E-Vac therapy can be delivered [26,27]. 

## Conclusions

BEF is a potentially dangerous complication during the course of esophageal malignancy or, even rarely, a complication of esophagectomy, carrying with it a high risk of morbidity and mortality. Its early detection is paramount for survival, and surgical repair can be lifesaving. Patients may present with nonspecific symptoms, primarily respiratory in nature and can be complicated by sepsis for a severe presentation or untreated, prolonged period. It is important to maintain a high index of clinical suspicion. Urgent surgical intervention has been shown to be superior to stenting in this disease's resolution and mortality prevention, but treatment should be discussed between the patient and physician reflecting the patient’s goals of care, involving the patient's desire for definitive versus palliative intervention. For patients who present with protracted, chronic symptoms, surgery is not urgent but may be considered after cooperative planning between the patient and the surgeon. Many may only request palliative care and in those instances supportive care and noninvasive management are preferred.

For obvious reasons, bronchoesophageal fistulas due to benign causes have a better prognosis than malignant ones. A multidisciplinary approach involving thoracic surgery, interventional pulmonology, gastroenterology, oncology, and a nutritionist is required to manage these complex cases. Simultaneous treatment of the fistula and the underlying etiology should be started as soon as possible. It is essential to focus on the patient's nutritional needs and aggressively treat superimposed infections. Supportive care should be offered to these patients, including cessation of oral intake, elevating the head to 45°, anti-reflux medicines, and enteral nutrition. Enteral nutrition may involve placement of a percutaneous endoscopic gastrostomy (PEG) tube, which delivers nutrition directly into the stomach, or a J-tube (jejunostomy tube), which delivers it into the small intestine (jejunum). Depending on the location of the fistula and the severity of the underlying condition, these patients can be considered for esophageal or bronchial stenting or both. Stenting can immediately palliate symptoms and increase the overall quality of life. Metallic stents are preferred over silicone stents for malignant fistulas. However, stenting is an option only for proximal lesions. Without treatment, the life expectancy of these patients is measured in weeks. Surgical intervention is not an option for these sick and terminal patients. Still, if their performance status is reasonable, palliative chemoradiation therapy can be considered.
